# The Endoscopic-to-Open Proximal Hamstring Repair Technique for Chronic, High-Grade Tears

**DOI:** 10.1016/j.eats.2025.103710

**Published:** 2025-07-17

**Authors:** Nicholas J. Lemme, Eric Y. Hu, Jesus E. Cervantes, Thomas E. Moran, Shane J. Nho

**Affiliations:** Section of Young Adult Hip Surgery, Division of Sports Medicine, Department of Orthopedic Surgery, Rush Medical College of Rush University, Rush University Medical Center, Chicago, Illinois, U.S.A.

## Abstract

Although open repair has been the historical standard for surgical treatment of proximal hamstring tears, enthusiasm for an endoscopic approach has grown because of its minimally invasive nature and advantages of improved visualization of the tendon footprint and adjacent neurovascular structures. Despite this, larger or more retracted tendon tears may still prove challenging to appropriately treat entirely endoscopically. The endoscopic-to-open technique aims to address this issue. Patients are positioned prone with the surgical limb placed on a padded Mayo stand with the knee flexed. Direct posterior and posterolateral portals are created in the gluteal fold for the endoscopic portion of the case, including ischial bursectomy, preparation of the tendon footprint, and anchor placement, before the interposing skin incision is created for the open portion of the case. The hamstring tendon is then accessed, mobilized, and repaired with a docking technique. Final arthroscopic visualization is subsequently performed to confirm adequate protection of critical neurovascular structures. An endoscopic-to-open treatment approach may provide surgeons with the visualization benefits of an endoscopic approach during preparation of the ischial tuberosity while also providing the access and maneuverability required for tendon mobilization and repair tensioning conferred by an open approach.

Hamstring injuries are one of the most prevalent lower-extremity injuries, with a prevalence around 30% among athletes.^1^ The most common hamstring injury experienced is a muscular strain at the myotendinous junction, which typically can be managed conservatively. Avulsions of the proximal hamstring are a rarer entity and occur mostly in sports involving explosive, forced flexion of the leg, such as soccer, sprinting, football, and water skiing. Depending on a variety of factors, different treatment options are recommended for proximal hamstring tears.

Treatment of more mild proximal hamstring tears or strains can be successfully achieved with conservative measures, including activity modification, anti-inflammatory medications, physical therapy, and biologic injections. When proximal hamstring tears involve 3 tendons or are retracted by greater than 2 cm or when patients do not improve with 6 months of conservative treatment, surgery may be required.^2^ Whereas open repair has been the historical standard for the surgical treatment of proximal hamstring tears, enthusiasm for an endoscopic approach has grown given its minimally invasive nature and the advantages of conferring improved visualization of the tendon footprint. In certain cases, an endoscopic-to-open treatment approach may be used, which provides surgeons with the visualization benefits of an endoscopic approach during preparation of the ischial tuberosity while also providing the access and maneuverability required for tendon mobilization and repair tensioning conferred by an open approach. The purpose of this article is to describe the senior author’s (S.J.N.) technique for endoscopic-to-open repair of proximal hamstring avulsion injuries.

## Surgical Technique

**Fig 1:**
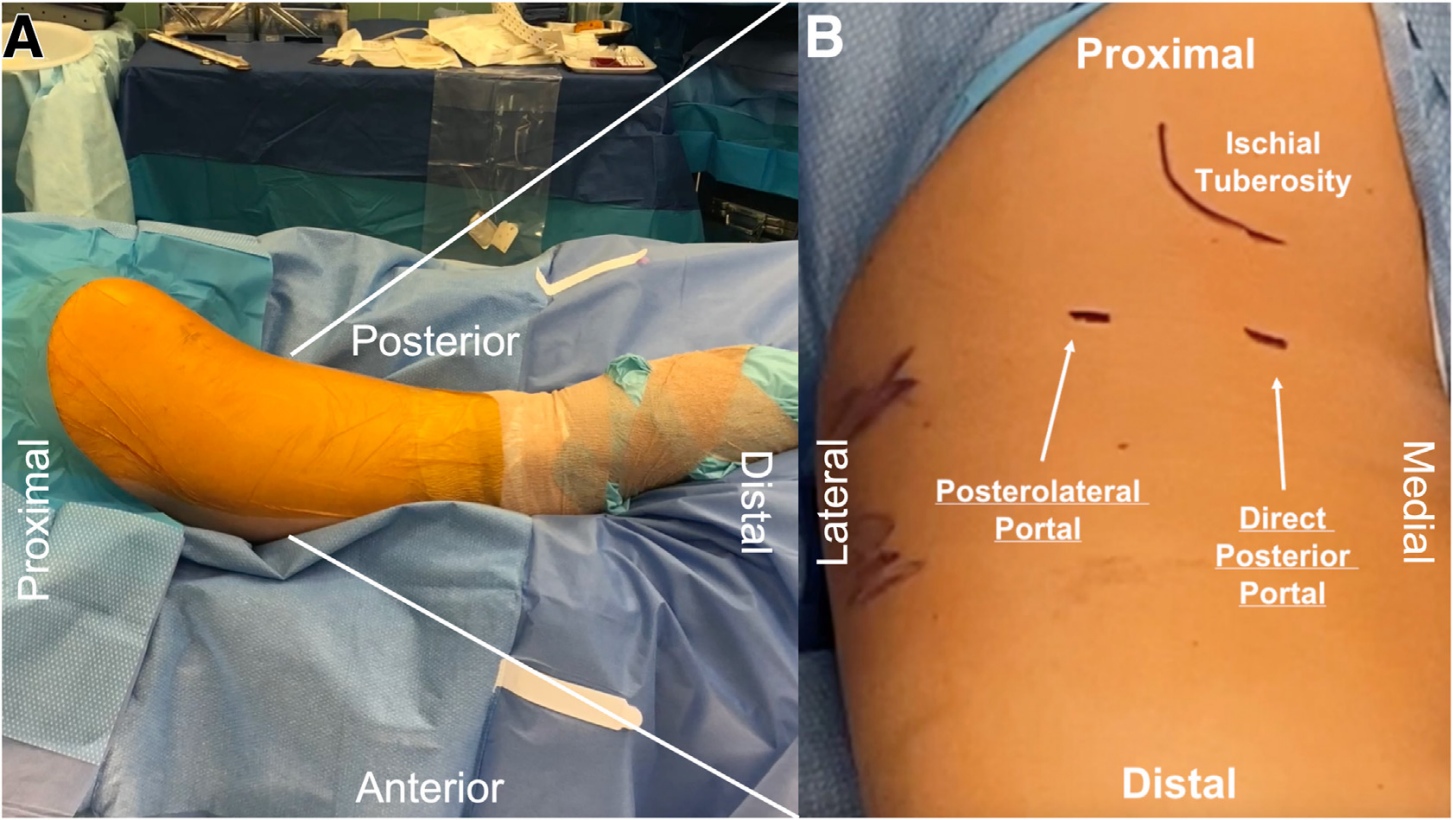
(A) The patient is placed in the prone position with the left surgical limb in hip extension and mild flexion of the knee to relax the gluteus maximus and tension on the sciatic nerve. (B) Relevant landmarks and portal placement for the endoscopic portion of the case are marked. The ischial tuberosity can be palpated. The direct posterior and posterolateral portals should be made along the gluteal crease. The direct posterior portal is made approximately 2 cm medial to the lateral aspect of the ischial tuberosity, and the posterolateral portal is created approximately 2 cm lateral to the lateral aspect of the ischial tuberosity. The leg can be extended to better identify this crease.

The patient is induced under general anesthesia with endotracheal intubation and then transferred onto the operating table in the prone position. C-arm fluoroscopy should be available to be introduced from the contralateral side of the operating room table. After preparation and draping of the surgical site in a sterile fashion, a padded Mayo stand is used to position the surgical limb with the hip in extension and the knee in flexion (Fig 1). This positioning takes tension off the hamstring muscle unit and sciatic nerve throughout the procedure.

**Fig 2:**
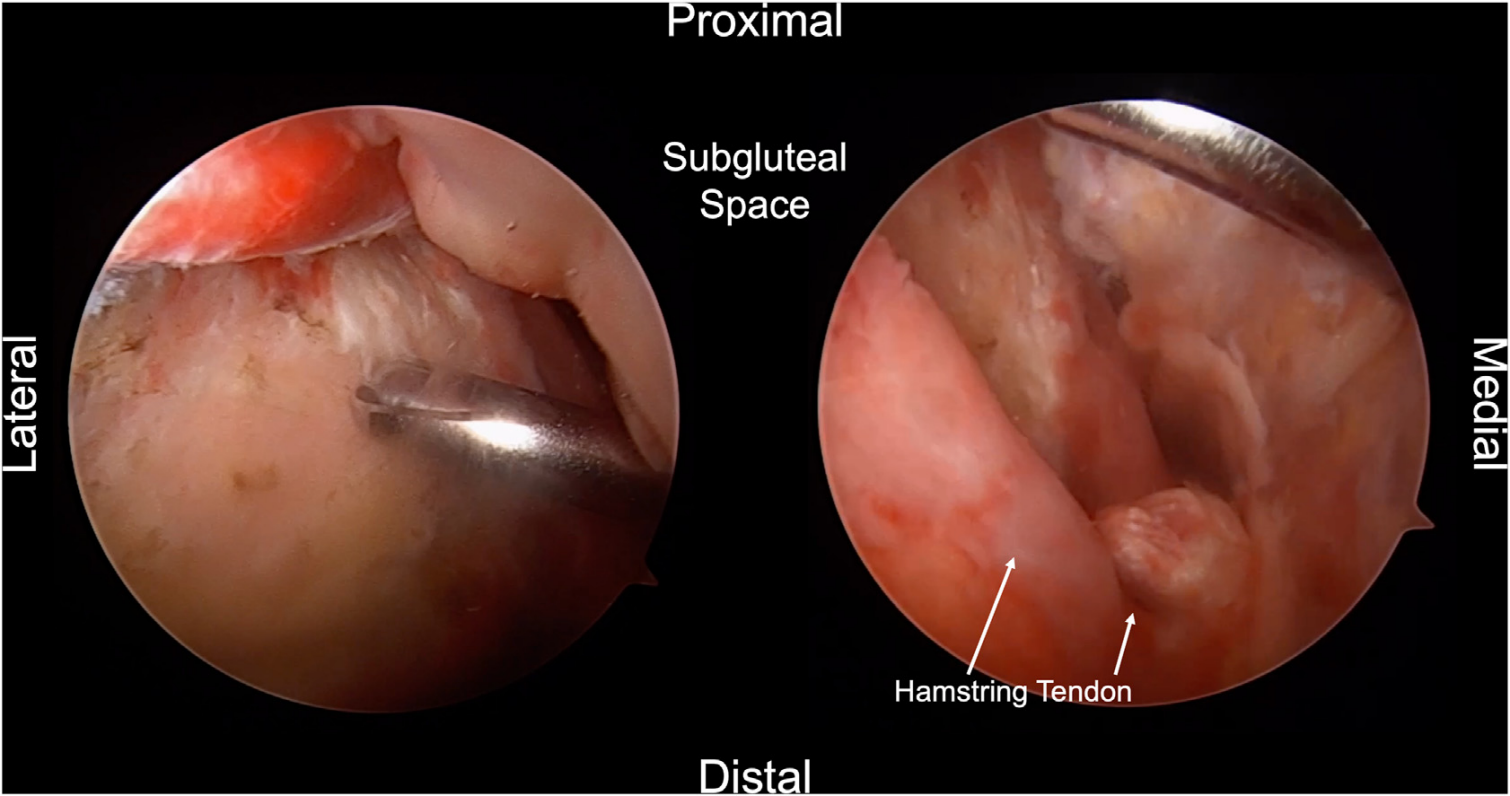
The arthroscope is placed in the posterolateral portal in the left surgical limb, and an arthroscopic shaver is used to perform an ischial bursectomy to improve visualization of the subgluteal space and surrounding neurovascular structures (left). It is important to insert both the arthroscope and shaver in the proper location within the subgluteal space prior to initiation of the bursectomy. After identification of the nerve, the stump of the proximal hamstring is visualized (right). An arthroscopic shaver is used to debride any adhesions surrounding the proximal hamstring stump and evacuate the surrounding hematoma. Proper evacuation of the hematoma and mobilization of the hamstring tendon stump are beneficial for manipulation of the hamstring during the open portion of the case.

**Fig 3:**
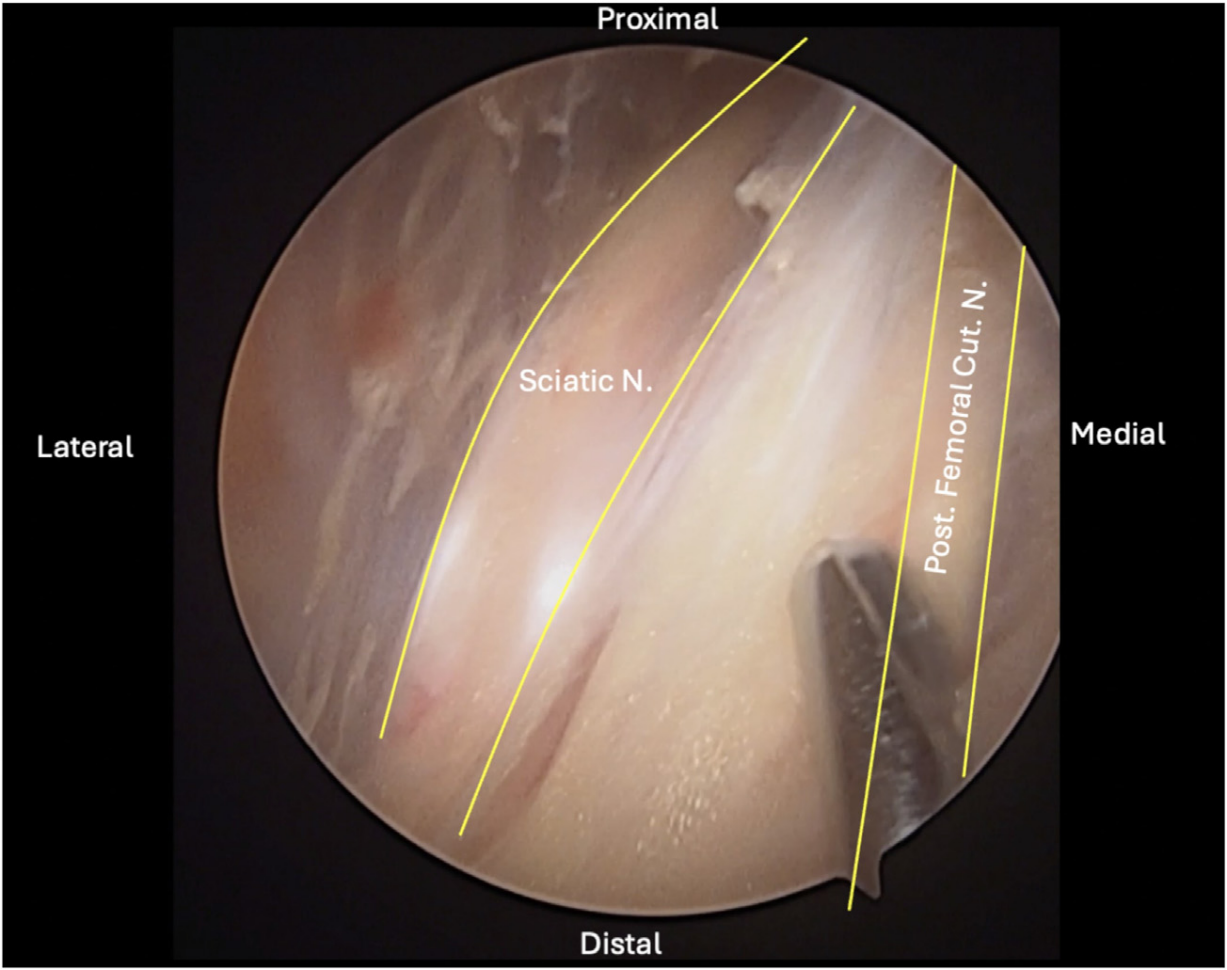
After debridement of the surrounding tissues, care should be taken to identify the neurovascular structures surrounding the left proximal hamstring. The sciatic nerve (Sciatic N.) and posterior femoral cutaneous nerve (Post. Femoral Cut. N.) are highlighted. The sciatic nerve lies more deep to the posterior femoral cutaneous nerve and lateral to the ischial tuberosity. The tissue surrounding the nerve should be gently debrided with a shaver.

**Fig 4:**
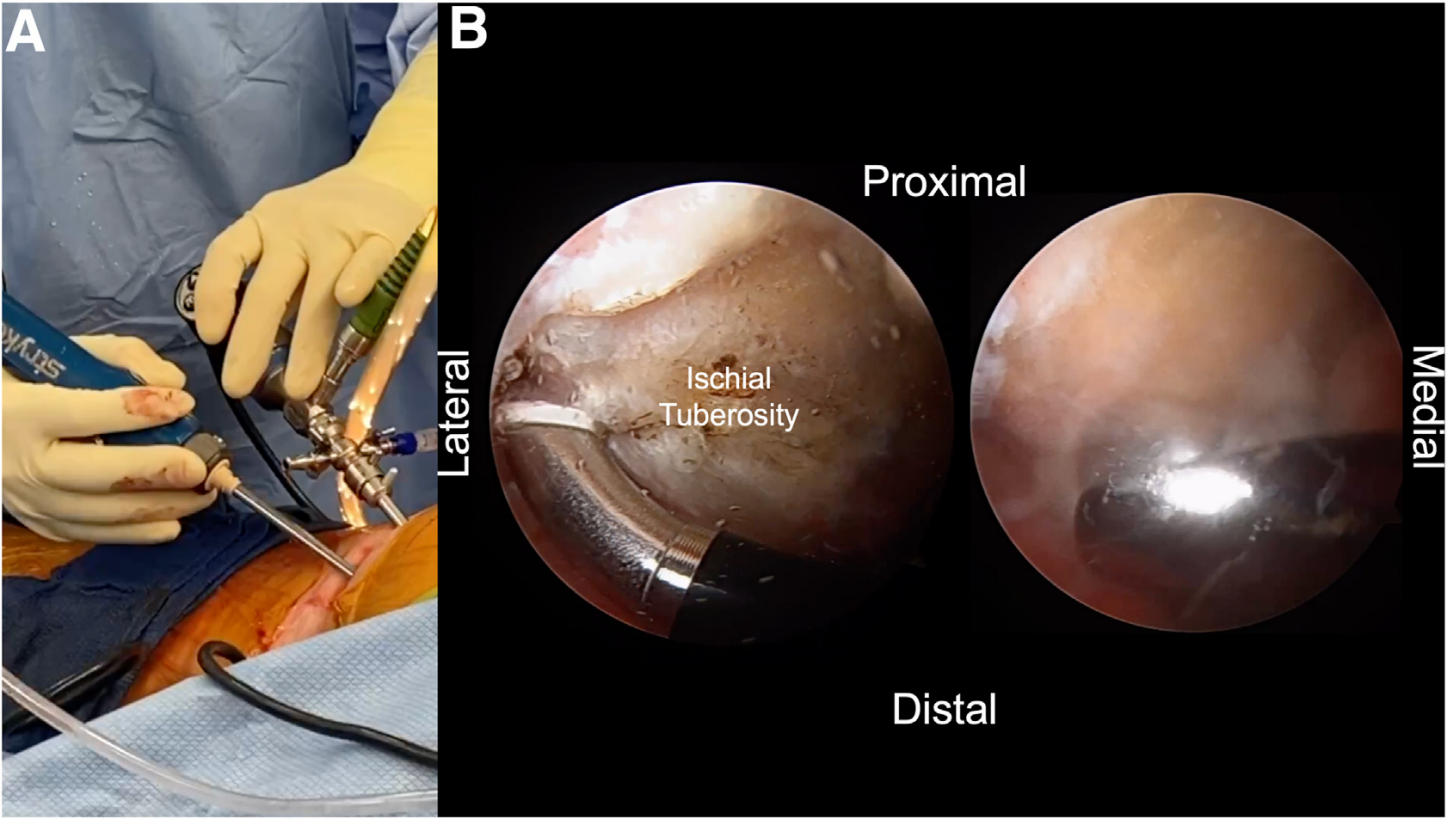
The left ischial tuberosity footprint is identified arthroscopically and cleared with radiofrequency ablation (A), followed by decortication with an arthroscopic burr (B). Tuberosity site preparation should be performed with the site for anchor placement in mind. Decortication is performed to provide a bleeding bed of bone to help augment the biological healing response.

**Fig 5:**
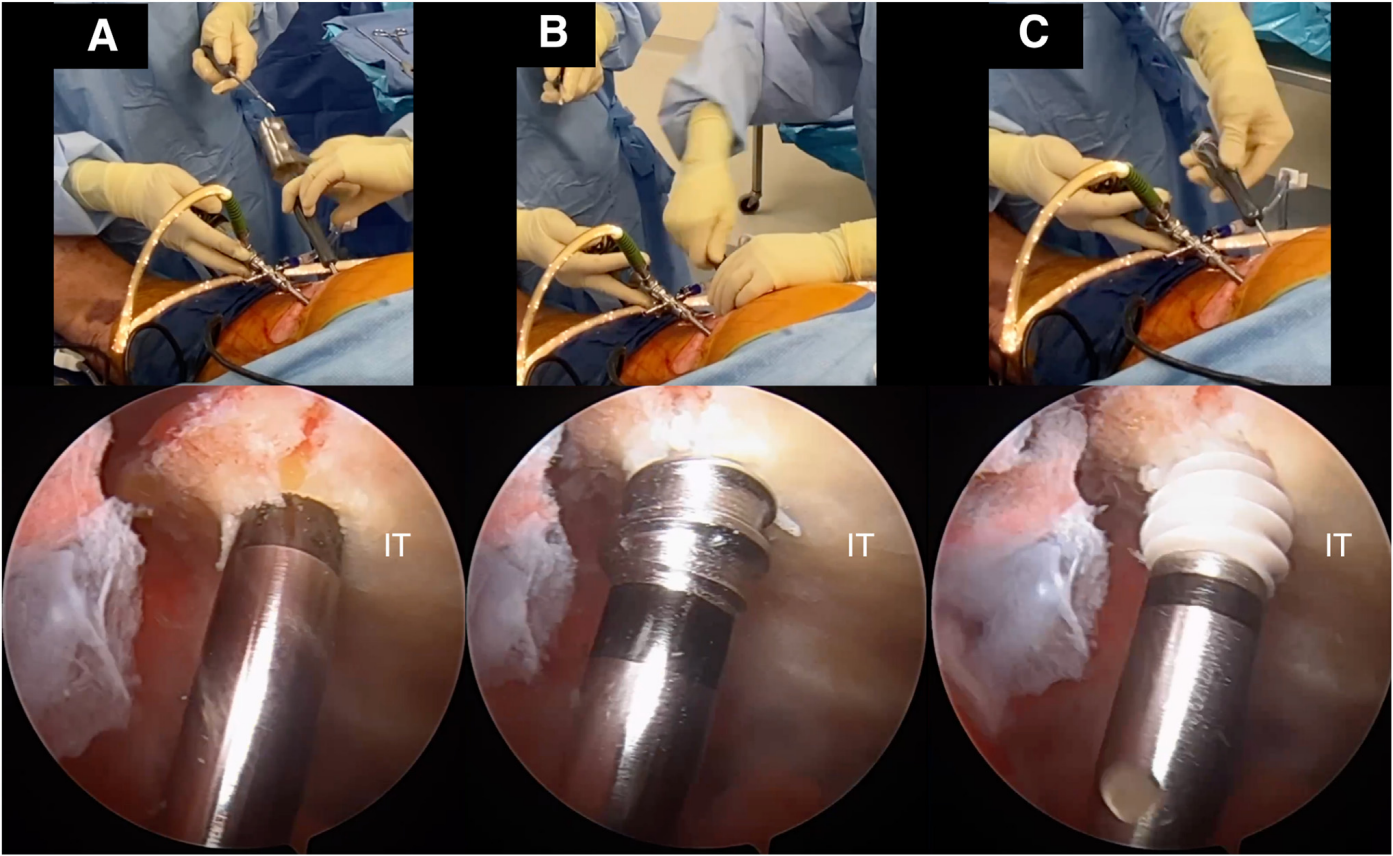
(A-C) Arthroscopic placement of a triple-loaded 5.5-mm PEEK (polyether ether ketone) AlphaVent anchor into the prepared left ischial tuberosity (IT). A punch (A) and tap (B) are used prior to placing the anchor. The anchor placement site is typically made in accordance with the hamstring tendon insertion sites on the ischium.

Two portals, the direct posterior and posterolateral portals, are placed into the gluteal fold during the combined open and endoscopic technique (Fig 1, Video 1). The direct posterior portal is made approximately 2 cm medial to the lateral aspect of the ischial tuberosity. After the introduction of the arthroscope, the posterolateral portal is created approximately 2 cm lateral to the lateral aspect of the ischial tuberosity under direct visualization. The portals are made with the intention of extending the skin incision between the 2 portals during the open portion of the procedure. After the arthroscope is switched to the posterolateral portal, an arthroscopic shaver (3.5-mm CrossBlade Smooth Bite Cutter; Stryker, Kalamazoo, MI) is used to perform an ischial bursectomy to improve visualization of the subgluteal space and surrounding neurovascular structures (Fig 2). The posterior femoral cutaneous nerve is typically identified first and is found lateral and distal to the lateral border of the ischial tuberosity. The sciatic nerve is typically found deep to the posterior femoral cutaneous nerve, ventral and lateral to the ischial tuberosity (Fig 3). The sciatic nerve should be identified and protected before any further preparation of the tendon footprint or tendon stump is performed. Once the posterior femoral cutaneous and sciatic nerves are identified, a radiofrequency ablation device (50-S Sweep Radiofrequency Ablation Probe; Stryker) may be used bluntly to expose the nerve. The proximal hamstring tear is then visualized (Fig 2). The arthroscopic shaver (3.5-mm CrossBlade Smooth Bite Cutter) is used to debride any adhesions surrounding the proximal hamstring stump and evacuate the surrounding hematoma. Radiofrequency ablation should be used during the bursectomy to maintain hemostasis. Preparation of the ischial tuberosity footprint is then achieved with radiofrequency ablation (50-S Sweep Radiofrequency Ablation Probe) to remove soft tissue before a 5.5-mm round arthroscopic burr (Formula 12-Flute Burr; Stryker) is used to decorticate the tendon footprint (Fig 4). Decortication of the footprint allows for the enhancement of the biological healing response of soft tissue to bone after the repair. Next, two 4.75-mm triple-loaded suture anchors (AlphaVent; Stryker) are placed from the posterolateral portal into the superolateral and inferolateral ischium along the decorticated footprint (Fig 5).

**Fig 6:**
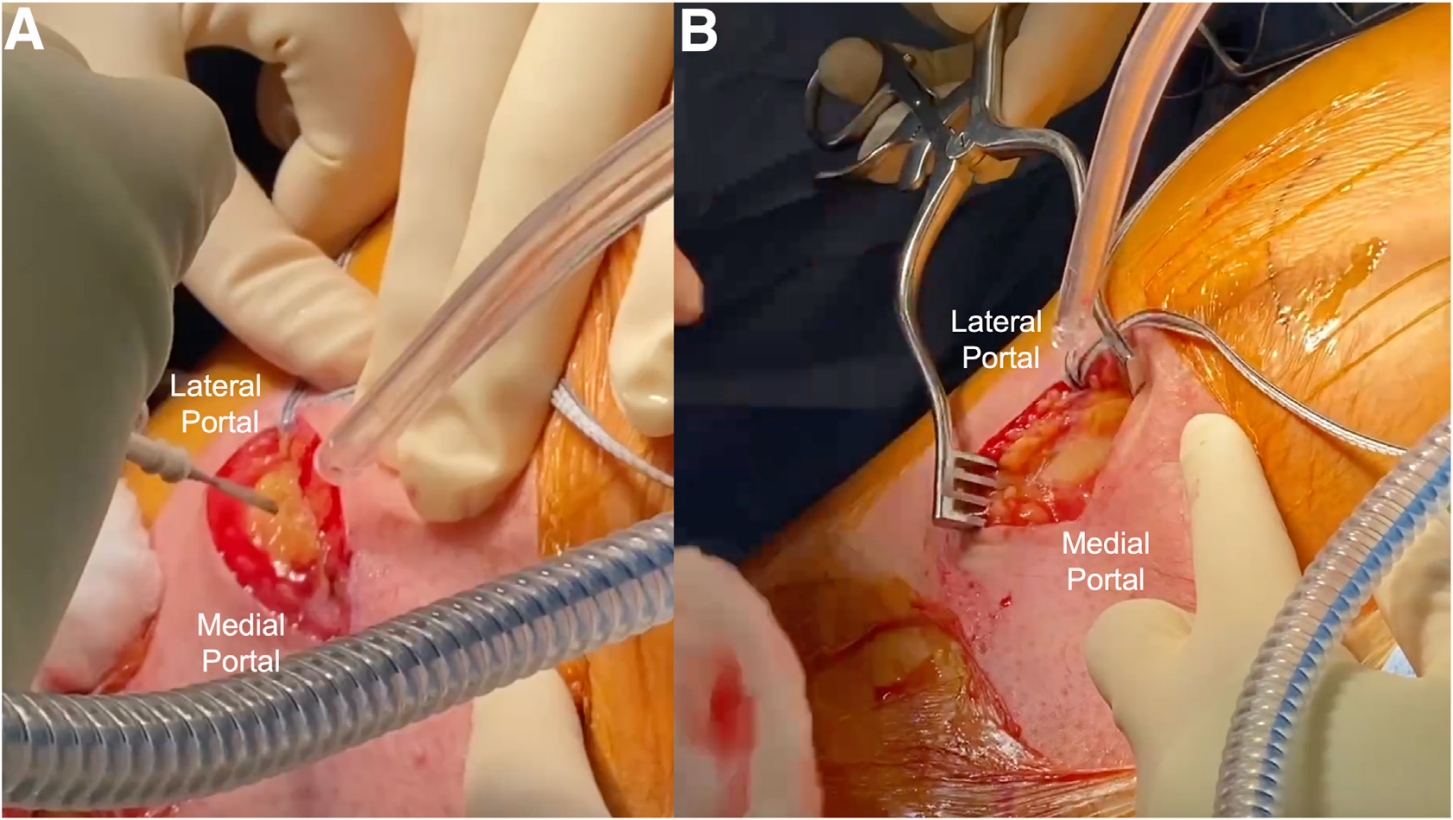
(A, B) An incision is created on the left hamstring by connecting the medial and lateral portals along the gluteal crease. If the portals are created too close to one another, extension of the incision can be performed to allow for manipulation of the hamstring tendon. The incision is taken down to the gluteal fascia. Once the fascia is incised, the gluteus maximus is retracted proximally to provide adequate exposure of the subgluteal space.

**Fig 7:**
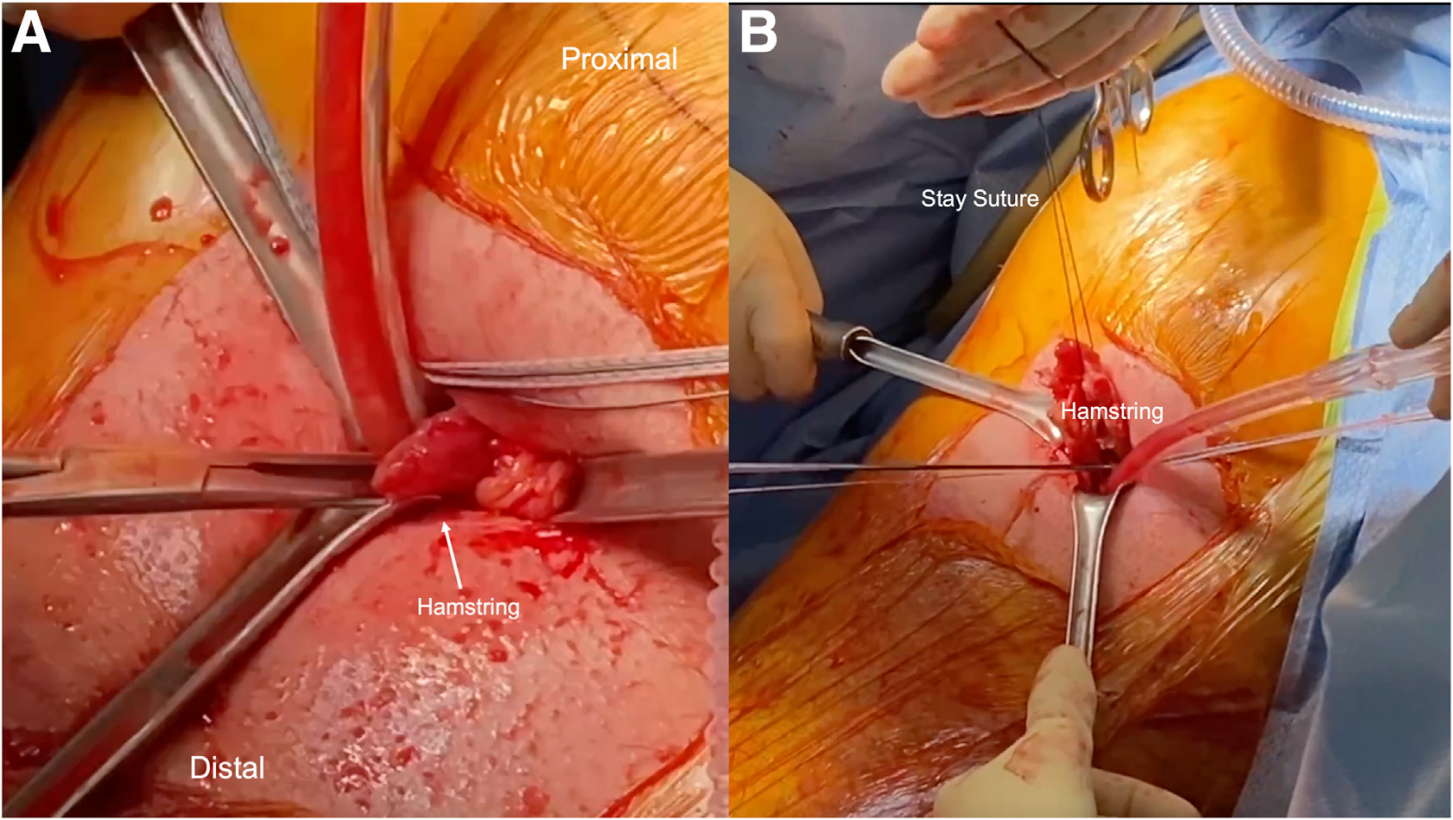
Identification and tagging of the left proximal hamstring are performed using a No. 1 Ethibond stay suture. (A) Blunt finger dissection may be used to identify the proximal hamstring. Deep retraction can be used for better visualization of the tendon during suture passing. (B) The stay suture is preferentially placed near the apex to allow for improved control and the ability of the tendon to be pulled superiorly during subsequent suture passing.

**Fig 8:**
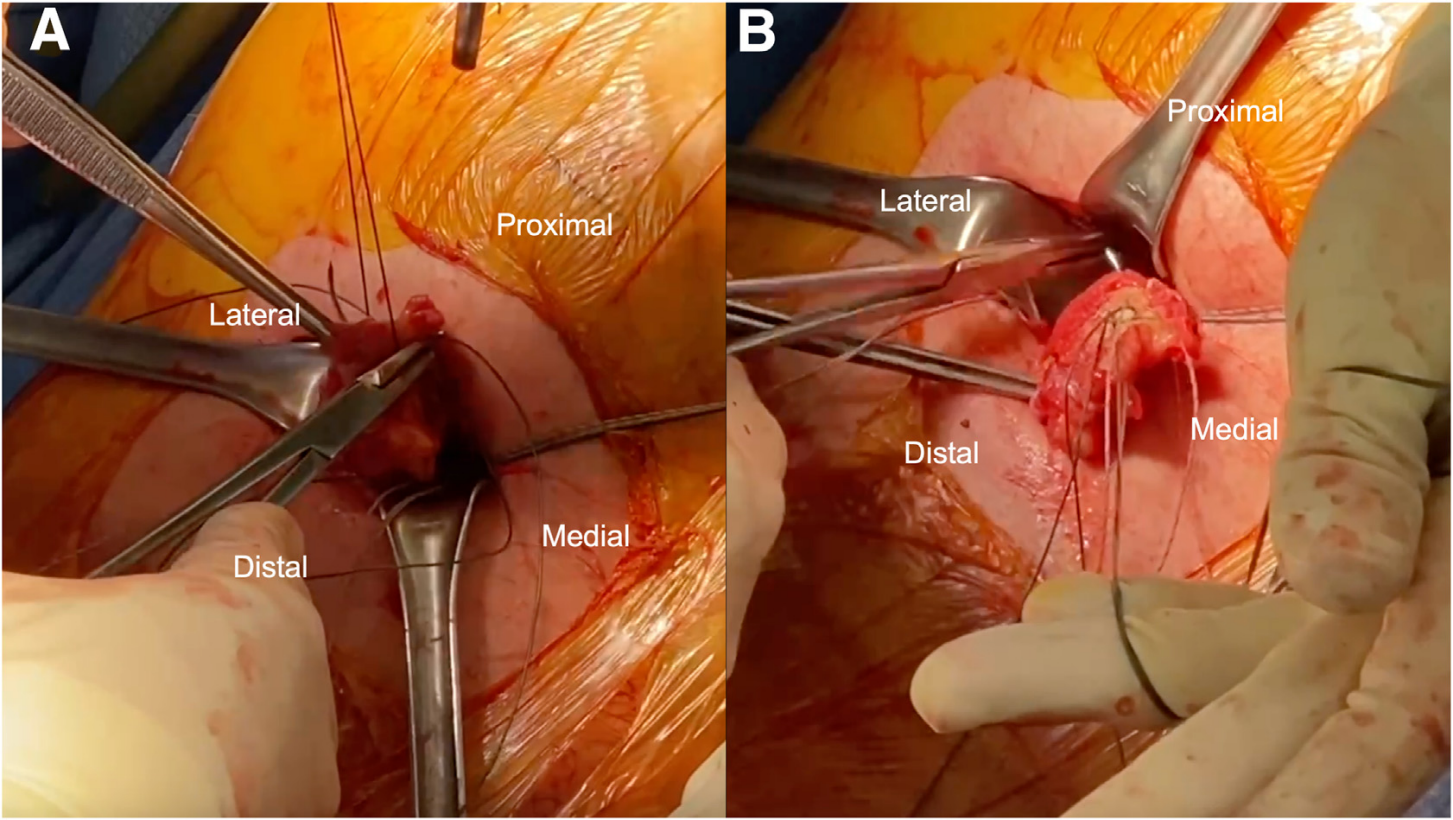
The pairs of triple-loaded sutures originating from each anchor are separated into the post and non-post limbs. Each of the non-post limbs is passed through the left hamstring stump in a running-locking fashion from proximal to distal on the lateral side of the tendon stump (A) and from distal to proximal on the medial side of the tendon stump (B), emerging superficially. The post limbs are passed through the tendon, exiting superficially to facilitate a tension-slide tendon repair. Tension is then applied to the post limbs to reduce the tendon to the ischial tuberosity while the knee is maintained at 40° to 60° of flexion. The non-post and post limbs of each pair of sutures from the anchors are tied sequentially.

To begin the open portion of the procedure, a skin incision is made between the direct posterior and posterolateral portals along the gluteal fold (Fig 6). The incision is carried down to the gluteal fascia. The fascia is then incised, and the gluteus maximus muscle belly is retracted proximally to reveal the posterior compartment of the thigh. With finger dissection, the proximal hamstring tendon stump is identified (Fig 7). Further deep retractors are placed to aid in the visualization of the hamstring stump. Once the tendon stump is identified, a No. 1 Ethibond stay suture (Ethicon, Somerville, NJ) is placed into the stump to allow for control of the tendon stump throughout the repair. Finger dissection is then performed circumferentially to the tendon to mobilize it proximally within the incision. The knee may be flexed to improve proximal delivery of the tendon out of the wound to facilitate the repair. The pairs of triple-loaded sutures originating from each respective anchor are then separated into the post and non-post limbs. The post limbs may be further identified with the use of a marking pen. Each of the non-post limbs is then passed through the hamstring stump in a running-locking fashion from proximal to distal on the lateral side of the tendon stump and from distal to proximal on the medial side of the tendon stump, emerging superficially (Fig 8). The post limbs are passed through the tendon, exiting superficially to facilitate a “tension-slide” tendon repair. Tension is then applied to the post limbs to reduce the tendon to the ischial tuberosity while the knee is maintained at 40° to 60° of flexion. The non-post and post limbs of each pair of sutures from the anchors are tied sequentially.

**Fig 9:**
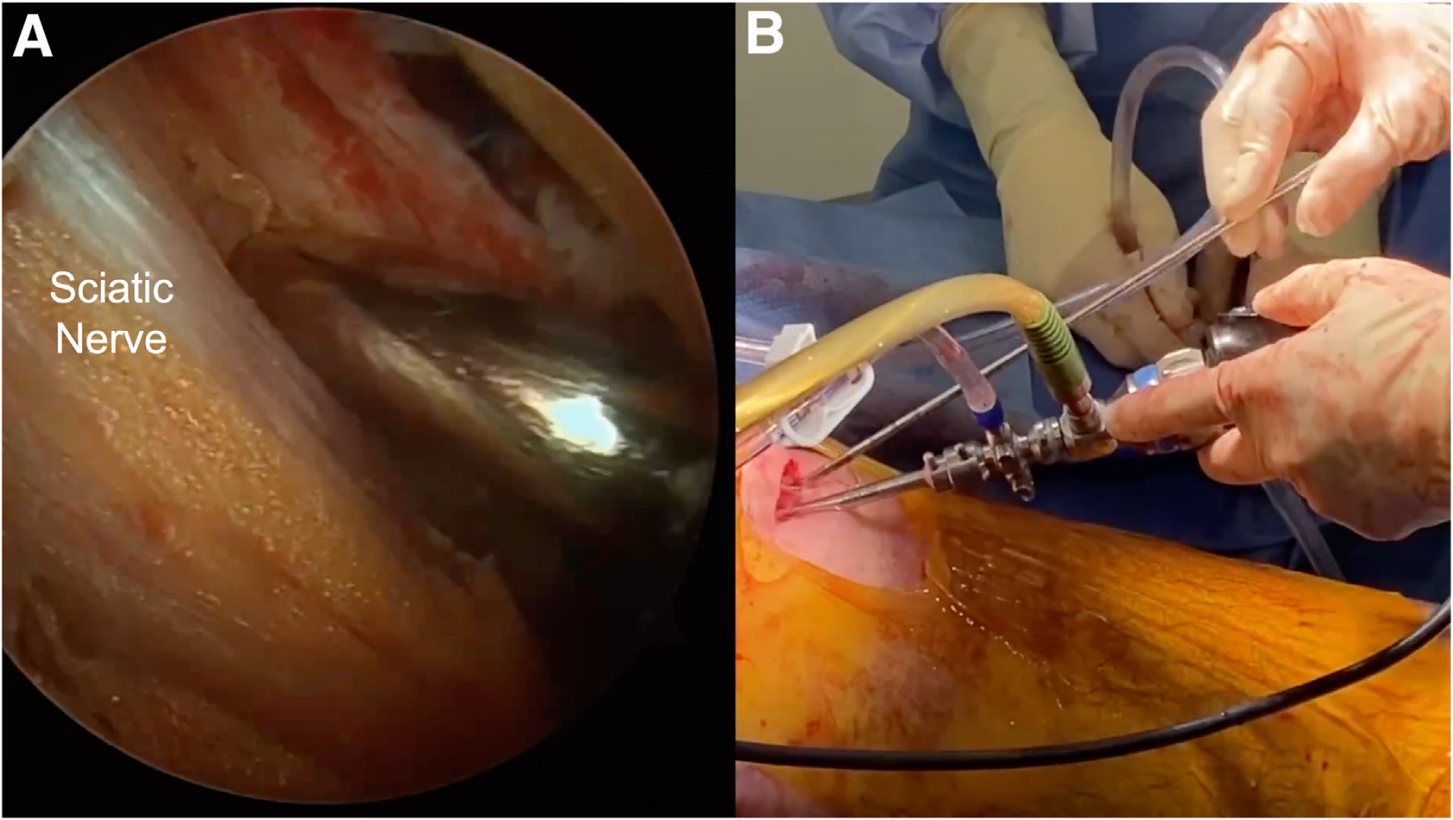
(A, B) Reintroduction of the arthroscope into the left subgluteal space allows for direct visualization of the repair. In addition, direct visualization of the nerve should be completed to ensure there is no undue tension, injury, or tether on the sciatic nerve. The incision site should be irrigated copiously, and soft massage can be beneficial in drawing out arthroscopic fluid prior to closure.

The arthroscope is reintroduced through the incision to perform final visualization of the repair and to ensure there is no undue tension or tether on the sciatic nerve (Fig 9). Once complete, the incision is copiously irrigated before a layered closure is performed. Skin closure is performed with absorbable suture and skin adhesive (Dermabond; Ethicon).

## Discussion

The presented technique describes an endoscopic-to-open approach to treat proximal hamstring tears. We recommend this approach in cases of more chronically retracted proximal hamstring tears that have failed greater than 6 months of conservative treatment. The pearls and pitfalls of this technique are highlighted in Table 1. Benefits of this approach include improved visualization of the neurovascular structures, ischial tuberosity, and retracted hamstring. Passing sutures through the tendon outside of the body also helps minimize the risk of nerve damage. Some disadvantages include a steeper learning curve for the endoscopic portion of the case, along with increased equipment costs required for both approaches (Table 2).

**Table 1:** Pearls and Pitfalls

Pearls
The use of an arthroscopic shaver, especially in cases with considerable adhesions, is imperative to identify and protect the sciatic nerve prior to conversion to an open view.
The open incision can be created by following the gluteal crease between the 2 arthroscopic portals.
Prior to conversion to an open approach, arthroscopic fluid should be extravasated from the working space to the greatest extent possible to improve visualization of the ischial tuberosity later during the case.
Careful hemostasis with radiofrequency ablation should be achieved during the endoscopic portion of the case to minimize fluid or hematoma accumulation, which may compress the nerve.
Pitfalls
The surgeon should be cautious with the pump pressure during the arthroscopic portion of the case. We recommend pressure between 30 and 40 mm Hg to prevent soft-tissue swelling and possible compartment syndrome while maintaining an optimal visual field.
The suture passing direction is important. The sutures should be passed from lateral to medial to avoid indirectly injuring the sciatic nerve.
Chronic injuries to the hamstring may present with more extensive adhesions to the neurovascular structures, making nerve identification and dissection more difficult.

**Table 2:** Advantages and Disadvantages

Advantages
The use of an arthroscope facilitates the visualization of the neurovascular structures, ischial tuberosity, and hamstring in cases of chronic tears.
The open portion of the case facilitates the manipulation of the hamstring in situations of extensive retraction, which would be more difficult to address via a solely endoscopic approach.
The open incision can be made smaller because the anchors are placed endoscopically.
Passing suture through the hamstring outside of the body helps minimize sciatic nerve injury.
Disadvantages
A steeper learning curve is involved with the endoscopic repair portion of the case.
A higher cost is incurred owing to the equipment required to perform both an endoscopic repair and an open repair.

Proximal hamstring injuries represent a common lower-extremity injury. Whereas open repair of proximal hamstring injuries has been the historical standard for patients traditionally indicated for surgical intervention, advancements in surgical technique have helped to expand clinical indications to include chronic partial-thickness tears that are recalcitrant to conservative management.^3^ Likewise, an endoscopic approach for the repair of proximal hamstring injuries has been increasingly described for the surgical treatment of complete proximal hamstring avulsion injuries. Surgeons favoring an endoscopic approach for proximal hamstring repair cite its minimally invasive nature and improved visualization of the tendon footprint and adjacent neurovascular structures as advantages over traditionally described techniques for open repair. An endoscopic approach is therefore more frequently used for partial avulsion injuries, for more acute tears with less retraction (<5 cm), or when the tendon is difficult to access underneath the gluteus maximus.^4^ Despite these advantages, an open approach remains more commonly used for larger avulsions or more chronically retracted tears because this may allow surgeons to better mobilize the torn and retracted tendon. This approach also allows the surgeon to use Krackow-type running and locking suture configurations in the repair construct that yield a biomechanically stronger or more durable repair than mattress-based single- and double-row repair constructs.^5^, ^6^, ^7^ The open approach is performed for larger, more chronic or more retracted tears (>5 cm) and for tears that require graft augmentation. An endoscopic-to-open treatment approach may therefore provide surgeons with the visualization benefits of an endoscopic approach during preparation of the ischial tuberosity while also providing the access and maneuverability required for tendon mobilization and repair tensioning conferred by an open approach.

Clinical outcomes have yet to be described for a series of patients undergoing our endoscopic-to-open approach for repair of proximal hamstring tendon avulsion injuries. Prior studies examining the endoscopic approach and the open approach in isolation have shown positive patient outcomes, high return-to-sport rates, and high satisfaction rates at short- and mid-term follow-up postoperatively.^4^^,^^8^, ^9^, ^10^, ^11^ Although further prospective and comparative studies are needed for validation of our technique for endoscopic-to-open repair of proximal hamstring tendon avulsion injuries, this technique may confer benefit to surgeons familiar with both techniques to address proximal hamstring injuries that exist outside the traditional relative indications for an endoscopic or open repair alone.

## Disclosures

The authors declare the following financial interests/personal relationships which may be considered as potential competing interests: S.J.N. reports a consulting or advisory relationship with Stryker and reports board membership with American Orthopaedic Society for Sports Medicine and Arthroscopy Association of North America. All other authors (N.J.L., E.Y.H., J.E.C., T.E.M.) declare that they have no known competing financial interests or personal relationships that could have appeared to influence the work reported in this paper.

## Supplementary Data

Video 1 Main steps of surgical procedure. The patient is positioned prone with the left surgical limb placed on a padded Mayo stand with the knee flexed. Direct posterior and posterolateral portals are created in the gluteal fold for the endoscopic portion of the case, including ischial bursectomy, preparation of the tendon footprint, and placement of two 4.75-mm triple-loaded suture anchors. Then, the interposing skin incision is created for the open portion of the case. The hamstring tendon is accessed, mobilized, and repaired with a docking technique. Final arthroscopic visualization is then performed to confirm adequate protection of critical neurovascular structures.
